# Skipping rope and pamphlet intervention to promote physical activity among young adolescents in South Africa: study protocol for a randomized controlled trial

**DOI:** 10.1186/s13063-026-09752-x

**Published:** 2026-05-11

**Authors:** Elisabetta Ferrero, Jabulani Ncayiyana, Sphindile Machanyangwa, Uttara Partap, Sachin Shinde, Shraddha Bajaria, Nathan Sivewright, Christine Neumann, Mary Mwanyika Sando, Michael Laxy, Till Bärnighausen, Wafaie Fawzi, Jacob Burns

**Affiliations:** 1 Department of Global Health and Population, Harvard T. H. Chan School of Public Health, Boston, USA; 2 School of Nursing and Public Health, University of KwaZulu-Natal, Durban, South Africa; 3 Africa Academy for Public Health, Dar Es Salaam, Tanzania; 4 Technical University of Munich, Munich, Germany; 5 Heidelberg Institute of Global Health, Heidelberg University Hospital, Heidelberg, Germany; 6 Department of Nutrition, Harvard T. H. Chan School of Public Health, Boston, USA; 7 Department of Epidemiology, Harvard T. H. Chan School of Public Health, Boston, USA

**Keywords:** Physical activity, Skipping rope, Informational pamphlet, Young adolescents, Community, South Africa

## Abstract

**Background:**

The World Health Organization (WHO) recommends that adolescents engage in at least 60 min of moderate-to-vigorous physical activity (MVPA) daily across the week to support healthy growth and development. Despite this guideline, physical inactivity is prevalent among adolescents in South Africa. This is concerning given the increasing burden of overweight and obesity. While nutrition is a critical driver of these conditions, physical activity represents a complementary and scalable strategy to improve overall adolescent health. This study aims to assess the effect of providing a skipping rope and an informational pamphlet to adolescents aged 10–14 years in South Africa on their physical activity intentions and behaviors.

**Methods:**

This study is a two-arm parallel-group, individual 1:1, unblinded randomized trial, nested within the Design and Evaluation of Adolescent Health Interventions and Policies in Sub-Saharan Africa (DASH) project. The intervention consists of providing adolescents 10–14 years old in a community setting in South Africa with a skipping rope and a pamphlet with general instructions for rope skipping and messages emphasizing the importance of regular physical activity. About 700 adolescent boys and girls are enrolled as part of this study. The intervention was administered during the first wave of data collection for the DASH study, or baseline, and outcomes will be assessed at both baseline and endline (12 months), during the second wave of data collection for DASH. As a primary outcome, the trial will test whether providing skipping ropes and pamphlets to intervention participants will increase their amount of weekly MVPA over 12 months, compared to participants in the control group, who will receive no materials. Secondary outcomes will be intention to do physical activity, assessed toward the end of the baseline interview, and meeting the WHO recommendations for physical activity, assessed at endline. The physical activity questionnaire was developed from the Physical Activity Questionnaires for Adolescents.

**Discussion:**

Improving physical activity behaviors and reducing sedentary time among adolescents are critical for fostering healthy lifestyles into adulthood. Assessing the effectiveness of this intervention to enhance physical activity intention and behaviors in South Africa is particularly important given the increasing burden of overweight and obesity.

**Trial registration:**

ClinicalTrials.gov NCT06516549. Registered on July 17th 2024. https://clinicaltrials.gov/study/NCT06516549.

**Supplementary Information:**

The online version contains supplementary material available at 10.1186/s13063-026-09752-x.

## Administrative information

**Table:** 

Title {1}	Skipping rope and pamphlet intervention to promote physical activity among young adolescents in South Africa: study protocol for a randomized controlled trial
Trial registration {2a and 2b}	NCT06516549
Protocol version {4}	April 18th 2026 Version 4
Funding {4}	German Federal Ministry of Education and Research (BMBF)
Author details {5a}	^1^Department of Global Health and Population, Harvard T. H. Chan School of Public Health, Boston, Massachusetts, USA ^2^ School of Nursing and Public Health, University of KwaZulu-Natal, Durban, South Africa ^3^ Africa Academy for Public Health, Tanzania ^4^ Technical University of Munich, Germany ^5^ Heidelberg Institute of Global Health, Heidelberg University Hospital, Heidelberg, Germany ^6^ Department of Nutrition, Harvard T. H. Chan School of Public Health, Boston, Massachusetts, USA ^7^ Department of Epidemiology, Harvard T. H. Chan School of Public Health, Boston, Massachusetts, USA
Name and contact information for the trial sponsor {5b}	University of KwaZulu-Natal
Role of sponsor {5c}	The study sponsor has oversight over study activities, including recruitment and data collection and management. The sponsor will work with the collaborators for data analysis and interpretation, and for writing and publishing the report

## Introduction

### Background and rationale {6a}

Adolescence, spanning ages 10–19 years, is a critical developmental period marked by significant physical, psychological, and social changes. During this time, adolescents have unique health and nutrition needs and are particularly vulnerable due to hormonal changes and growth spurts [1]. It is also a phase when behaviors can easily be influenced and modified, making it an opportune time to address behaviors that impact long-term health and well-being [2]. With adolescents comprising about 16% of the world population and 23% of the population in sub-Saharan Africa (SSA) [3], addressing their health needs is critical to ensure their present well-being, as well as foster healthier life trajectories.

Physical activity is a key lifestyle behavior of a healthy life. Research shows that regular physical activity improves several physical and mental health outcomes among adolescents, including muscular strength and flexibility, body composition, bone density, cardiovascular health, mood, sleep quality, academic performance, and social behaviors [4, 5]. The World Health Organization (WHO) recommends at least 60 min of moderate-to-vigorous physical activity (MVPA) daily across the week for adolescents, with vigorous activities at least 3 days per week, while minimizing sedentary time [6]. MVPA can include brisk walking, jogging, and cycling, while vigorous activities include running, swimming, aerobic dancing, and jumping rope.

In SSA, adolescents tend to be inactive, with about 82–90% of adolescents reporting low physical activity levels in recent surveys [7]. In South Africa, a significant proportion of adolescents fail to meet the physical activity recommendations, with about 93% of males and 95% of females recently reporting low physical activity outside of school or sports clubs [8], and only 50% of children and adolescents meeting the recommended 1 h of MVPA per day [9]. This widespread physical inactivity among adolescents poses a significant public health threat in South Africa, where about 21% of adolescents are overweight and obese [10].

Skipping, or jumping rope, is a simple physical activity that engages the whole body and can contribute to MVPA depending on pace and skill level, and can be easily promoted among adolescents. It has been shown to improve cardiorespiratory fitness and bone density [11, 12], and is a popular type of physical activity and recreational tool among youth in many SSA countries, including South Africa [13]. A randomized controlled trial from China conducted with middle school students found that providing a 12-week jump-rope intervention as an afterschool program was associated with significant improvements in muscle strength, bone mineral density, and flexibility [11]. Additionally, a recent randomized controlled trial from Saudi Arabia, recruiting adolescents aged 12–18 with obesity from both schools and community settings, found that jumping rope was associated with a significant reduction in BMI, an increase in lean body mass, and improvements in muscle tone [14].

While there is substantial evidence of short-term positive and successful implementation of skipping rope in schools [11, 12, 15–17], fewer studies have explored the effect of skipping rope through community settings outside of school, such as religious and youth centers, and little is known about the effect of longer-term interventions, especially in South Africa. Additionally, to our knowledge, there is limited evidence on skipping rope interventions specifically targeting younger adolescents (aged 10–14 years) in South Africa and other SSA settings. The many physical, social, emotional, and cognitive changes that influence decisions and behaviors among younger adolescents make this age period especially important to shape healthy behaviors early on [18].

To address these gaps, a randomized controlled trial is being implemented to promote physical activity among adolescents aged 10–14 in a community setting in South Africa. The intervention involves providing adolescents with a skipping rope and an informational pamphlet on rope skipping and the benefits of physical activity, as pamphlets have been successfully utilized in previous research to promote physical activity among adolescents [19] and can be easily distributed to all participants. The study will provide important evidence on the benefits of this intervention for both in- and out-of-school adolescents. The trial will assess changes in physical activity intentions and behaviors over 12 months. The trial is part of the Design and Evaluation of Adolescent Health Interventions and Policies in Sub-Saharan Africa (DASH) project [20], aimed at boosting adolescent health in the SSA region through rigorous population-based intervention and policy research. Specifically, it will be nested within the longitudinal data collection activities of DASH and will thus be less resource-intensive compared to traditional intervention trials.

### Objectives {7}

The study aims to investigate the effect of providing a skipping rope and an informational pamphlet with physical activity information (intervention group) on physical activity intentions and behaviors among young adolescents in South Africa, compared to not providing these materials (control group). We hypothesize that providing the skipping rope and the informational pamphlet about physical activity to adolescents may be more effective in improving their levels of MVPA at 12 months after baseline assessment, compared to not providing these materials. The secondary hypotheses are that providing the intervention will also a) increase the number of adolescents who intend to engage in physical activity over the 7 days following the interview, at baseline assessment; and b) increase the proportion of adolescents who meet the WHO MVPA recommendations at 12 months after baseline assessment, compared to not providing these materials.

### Trial design {8}

This study is a two-arm parallel-group, individual 1:1, unblinded randomized trial nested within the DASH cohort study [20, 21], i.e., a “randomized trial-within-cohort.” Wave 1 of the DASH cohort study, which took place between October 2024 and February 2025, served as the trial baseline. The intervention (skipping rope and pamphlet) was provided towards the end of the baseline interview, after all skipping rope-related outcome questions had been completed and before the question on intention to engage in physical activity was asked. Wave 2 of the DASH cohort study, 12 months after, will serve as the trial endline. Changes in intention to do physical activity will be assessed at baseline, while changes in physical activity behaviors will be assessed at endline. The trial is based on a superiority framework, as it will test the superiority of providing the skipping rope and pamphlet to adolescents for physical activity, compared to not providing the materials.

**Fig. 1:**
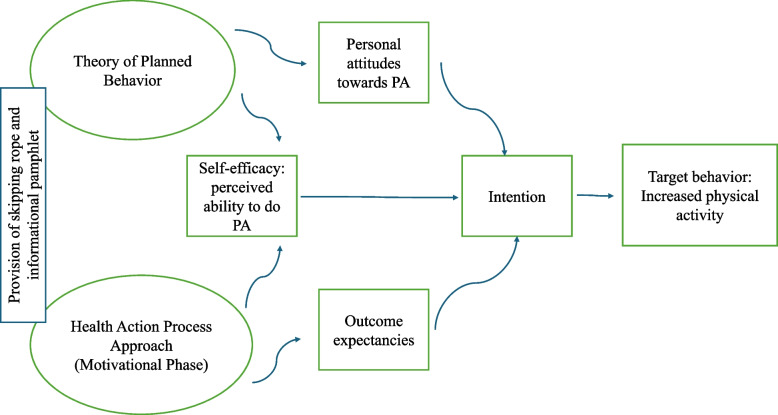
Theoretical framework for skipping rope intervention

This intervention is based on the Theory of Planned Behavior, which suggests that a person’s health behavior is determined by their intention to perform the behavior [22]. This theory assumes that three types of beliefs affect a specific behavior, which in this intervention would be physical activity: attitude toward physical activity, perceived attitudes of peers and influences toward physical activity, and perceived ability to do physical activity. The intervention is also based on the Health Action Process Approach [23], which proposes that behavior change follows a motivational phase, forming intentions, and a volitional phase made of planning and acting. The motivational phase is influenced by task self-efficacy, outcome expectancies, and risk perception [23]. Within this framework, the intervention aims to potentially influence adolescents’ attitudes toward physical activity, outcome expectancies, and self-efficacy through the provision of information and a low-cost skipping rope, thereby supporting intentions and engagement in physical activity. Figure 1 illustrates how these two theoretical frameworks are used to structure and interpret the potential pathways of influence in the intervention.

## Methods: participants, interventions, and outcomes

### Study setting {9}

This trial is embedded within the DASH cohort study [20], through the Umlazi Health and Demographic Surveillance System (HDSS), also called USINGA (Umlazi Surveillance Initiative to Nurture Grassroots Action), in Durban, South Africa. This HDSS operates as a standardized and comprehensive field-based information system and research platform. It collects prospective data from entire populations, including adolescents, at both individual and household levels, from communities facing developmental constraints. The USINGA HDSS covers two wards with a combined population of 80,550 individuals residing in 21,012 households of formal dwellings and 7698 households of informal dwellings. The HDSS is divided into 3 zones with each zone further divided into 5 blocks [24]. As part of the DASH cohort study, a random sample of 2000 individuals aged 10–24 was drawn from one ward of the USINGA HDSS. After the initial sampling of 2000 adolescents, resampling occurred until a full 2000 participants were included in the DASH cohort study. Of these 2000 individuals, all those aged 10–14 were invited to participate in this randomized trial.

### Eligibility criteria {10}

#### Inclusion criteria

Inclusion criteria for this study are as follows:

- Study participant is between ages 10 and 14 years;
- Given minors will be enrolled (i.e., less than 18 years of age): one of the parents or a guardian provides written informed consent;
- The minor participant provides informed assent; and,
- Study participant is a resident of the study area and intends to stay in the study area for the duration of the study.

#### Exclusion criteria

The following exclusion criteria are applied to the study:

- Participants who do not meet the above-listed criteria;
- Participants with physical disabilities that, based on individual assessment and available information, would make participation in moderate physical activity unsafe or not feasible;
- Those participants whose capacity to make meaningful decisions is in question because they are “cognitively impaired”;
- Individuals with communication difficulties; and,
- Study participants who report suicidal behaviors during baseline data collection.

### Who will take informed consent? {26a}

Trained fieldworkers obtained informed consent from all individuals. For all minors, informed consent from one of the parents or a guardian and informed assent from the individual were required. The confidentiality of participants’ information was clearly described in the consent script. The consent script explained that no personal identifiers will be used, and participation is completely voluntary. The script specified that an individual may refuse to participate or discontinue the study at any point without penalty, and without influence on any aspect of their lives. It will also discuss the risks and benefits of their participation in the study. It was made clear to individuals that they may ask questions about the study and their participation at any time.

### Additional consent provisions for collection and use of participant data and biological specimens {26b}

NA: There are currently no plans to collect and use data and specimens in ancillary studies.

## Interventions

### Explanation for the choice of comparators {6b}

The intervention group received a skipping rope and an informational pamphlet with instructions on how to use the skipping rope and information on the benefits of physical activity. The control group received no intervention. No intervention was selected for the control group, as there is no clear minimum level of “standard care” that can be delivered for interventions related to physical activity. Furthermore, the research team was interested in understanding the effect of providing simple resources for physical activity over and above currently available resources in the community.

### Intervention description {11a}

The intervention consists of a skipping rope provided to participants, along with a pamphlet containing information on how to use the skipping rope and general information about physical activity guidelines. The ropes are procured locally through the University of KwaZulu-Natal (UKZN). The pamphlet is trifold and provides instructions to skip rope, such as “Ensure the rope reaches nearly up to your shoulders when folded in half, step over the rope, so that it hangs behind you, swing the rope over your head, and, when the rope is coming towards the top of your feet, hop over it.”

Given that skipping rope is generally considered MVPA once a basic level of proficiency is achieved, and that the duration of skipping from previous interventions ranged substantially, including from 15 min once a week [16] to 45 min three times a week [11, 14], the pamphlet provides instructions to start skipping for 10–15 min every other day, and then 10–15 min every day once it becomes easier, to also prevent potential injuries due to over-exercising [25]. In addition to skipping rope, the pamphlet encourages adolescents to do physical activity for at least 60 min per day and provides examples of other activities, warm-ups, and cool-downs, along with information on the benefits of physical activity and skipping rope. The pamphlet can be accessed through Additional file 1.

Wave 1 of the DASH cohort study will serve as the trial baseline. The provision of a rope and an informational pamphlet to the intervention group happened toward the end of the baseline interview. The fieldworker conducting the survey interview provided each participant in the intervention group with a rope and pamphlet and read a short script explaining how to use the rope, outlining the main benefits of physical activity for adolescents, and asking participants to read the pamphlet in their own time. On the other hand, the control group did not receive any intervention. Because this trial presents risks for contamination between intervention and control groups, fieldworkers instructed participants not to share their rope with others. In order to monitor any potential contamination, during endline, participants in the intervention group will be asked if they used the rope alone or with friends, and participants in the control group will be asked if a friend or neighbor shared a rope with them.

### Criteria for discontinuing or modifying allocated interventions {11b}

Participants may voluntarily withdraw from this trial at any time without providing a reason, in accordance with ethical guidelines. Additionally, if the participant discontinues the overall DASH cohort study, within which this trial is nested, the participant will also be withdrawn from the trial.

### Strategies to improve adherence to interventions {11c}

To improve adherence to the intervention, fieldworkers provided participants in the intervention arm with instructions on how to use the skipping rope, allowing them time to practice and get comfortable skipping. Fieldworkers also explained the health benefits associated with physical activity and distributed a pamphlet reinforcing key messages on the importance of physical activity and the specific benefits of skipping rope.

### Relevant concomitant care permitted or prohibited during the trial {11d}

NA: Any concomitant care will not interfere with the trial.

### Provisions for post-trial care {30}

NA: Post-trial care will not be provided as part of this trial.

### Outcomes {12}

The primary outcome will be total duration of MVPA per week (minutes of MVPA/week), measured at endline assessment (12 months after baseline). This has been chosen as the primary outcome, because the WHO recommends that adolescents engage in at least 60 min of MVPA daily, in addition to incorporating vigorous-intensity aerobic activities at least 3 days per week [6]. At baseline and endline, participants are asked about the duration and frequency of MVPA they engaged in throughout the week preceding the interview, in- and out-of-school. Relevant questions have been readapted from the Physical Activity Questionnaire for Adolescents (PAQ-A) [26], and the International Physical Activity Questionnaire for Adolescents (IPAQ-A) [27]. The duration of MVPA both in- and out-of-school will then be calculated by multiplying the minutes of MVPA reported for the most recent day of physical activity by the number of days MVPA was performed. To determine the total weekly duration of MVPA for each participant, the duration in minutes of MVPA performed in-school and out-of-school will be summed.

Secondary outcomes will be (a) intention to do physical activity in the coming week, compared to the 7 days preceding the interview, between the intervention and control groups, measured at baseline after the intervention materials are provided; and (b) meeting the WHO MVPA recommendations (> = 420 min/week) over the 7 days preceding the interview at endline. Finally, at endline, the trial will assess the following process outcomes: (a) whether participants in the intervention group have been using the rope alone or with friends; and (b) whether a friend or neighbor shared a skipping rope with participants in the control group. This will allow the investigators to identify any potential contamination.

### Participant timeline {13}

The skipping rope intervention was administered by fieldworkers during the first round of data collection in the DASH cohort study (Wave 1 of DASH in 2024) and took approximately 10 min. Baseline outcome assessment will occur during this first round of data collection; endline outcome assessment will occur during the second round of data collection (Wave 2 of DASH), approximately 12 months later. Table 1 shows the study’s timeline.

**Table 1:**
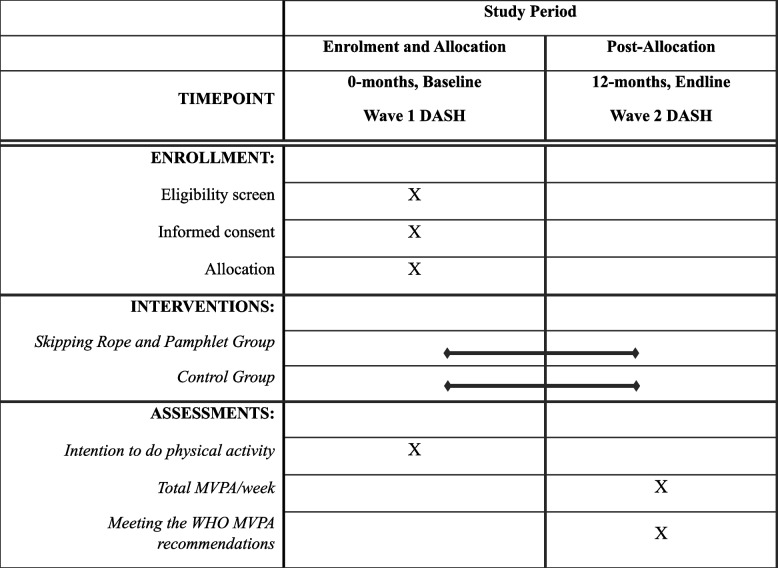
Study timeline for the skipping rope intervention

### Sample size {14}

For the overall DASH cohort study, a random sample of 2000 individuals aged 10–24 was drawn from existing population sampling frames created and maintained by the Umlazi HDSS in South Africa. Within DASH, after the initial sampling of 2000, resampling occurred until a full 2000 participants were included in the DASH cohort study. Of the 2000 individuals who consented to participate in the overall DASH study, all those aged 10–14 were included in this randomized trial.

#### Power calculation

We anticipate having 700 participants enrolled throughout the duration of the study (1:1 ratio between intervention and control groups). To calculate the statistical power for our intervention trial, we used a Stata menu-driven command to estimate power based on various scenarios for the changes in the primary outcome, total minutes of MVPA/week.

The total minutes of MVPA per week are based on previous research on physical activity for adolescents in South Africa and a few other countries [28–36]. Based on such research, the following assumptions were considered:

- Minutes of MVPA per week among adolescents in South Africa vary significantly. To be conservative, we assumed a baseline value of 250 min/week of MVPA across boys and girls, with a standard deviation (SD) of 160 min/week [28].
- 15% loss to follow-up [37, 38].

Based on these assumptions, the study will have 80% power to detect a minimum difference of 33.9 min/week in MVPA between the intervention and comparison arms (Table 2).

**Table 2:** Power calculation for MVPA outcome (*n* = 700 participants, 350 participants in each arm)

Baseline mean	Standard deviation	Minimum detectable difference in mean	Number of participants in each arm	Expected power
250	160	43.7	350	95%
250	160	39.3	350	90%
250	160	36.3	350	85%
250	160	33.9	350	80%
250	160	31.9	350	75%

The table shows that with a power of 80%, 350 participants in each arm will yield a minimum detectable difference of 33.9 min/week between the intervention and control groups at endline, with a baseline mean value of 250 min/week of MVPA and an SD of 160 min/week. The number of participants per arm may vary slightly

### Recruitment {15}

In the USINGA HDSS site, male and female residents aged 10–24 years were recruited for the overall DASH cohort study through household-based random sampling. The site used a sampling strategy that covered Ward 1. The sample was stratified by age and gender, with random selection used to ensure representative coverage of the community. Specifically for this trial, those participants of the DASH cohort study aged 10–14 who met the eligibility criteria were recruited. For the consent process, fieldworkers visited the selected households for 10–14 years old adolescents, who, by South African law, are considered minors. Written consent was obtained from a parent/guardian and if the parent/guardian consented, assent was obtained from the adolescent. The interview for data collection was conducted in the absence of a parent/guardian to ensure privacy.

## Assignment of interventions: allocation

### Sequence generation {16a}

As described above, this trial is nested within the DASH cohort study, which uses existing population sampling frames in its sampling strategy. Using a pre-defined list of participants for the study based on the information from the HDSS system, researchers used statistical computing software to define a random allocation sequence for all participants potentially eligible for this trial (i.e., those aged 10–14 years). This will be a simple 1:1 random allocation. The fieldworkers and others responsible for implementing the study in the community were not involved in the sequence generation.

### Concealment mechanism {16b}

The allocation was not concealed. However, all fieldworkers were instructed of the importance of treating the participant as indicated by the allocation sequence (i.e., implementing the intervention or control condition) and were prompted on the tool used for data collection to confirm that they have done so. Additionally, given that, in principle, all participants of the DASH cohort study between the ages of 10–14 years are eligible for the trial, this lack of concealment is very unlikely to lead to selective enrollment. The assignment to study arms was pre-decided by a researcher or group of researchers who are not involved in field implementation and was not decided by the fieldworkers.

### Implementation {16c}

The sequence was generated by researchers at the Technical University of Munich (TUM) in Germany, who are involved in the DASH cohort study but are not involved in the concrete implementation of this trial. The allocation was pre-loaded into the Open Data Kit (ODK) Collect software, which is used within the DASH cohort study for tablet-based digital data collection. This pre-loading ensured that the group assignment for each individual was correctly programmed on the tablet. It also ensured that this group assignment was clear to the fieldworker leading the data collection interview. The eligibility criteria were assessed by this fieldworker through standardized questions in the DASH cohort study survey. For each participant meeting the eligibility criteria, the fieldworker was walked through all steps of introducing and providing the intervention as well as assessing the outcome through instructions and scripts provided automatically on the tablet.

## Assignment of interventions: blinding

### Who will be blinded {17a}

Given the nature of the overall DASH cohort study and of this intervention, blinding is not possible. Intervention implementation and outcome assessment, both undertaken by the fieldworker, will be standardized within the tablet-based collection, using concrete instructions and scripts. The study analyst will be blinded until after completion of data analysis. The process of blinding will be undertaken by the Africa Academy for Public Health (AAPH), an independent organization registered in Tanzania and dedicated to addressing public health priority challenges in SSA, that will securely provide the data to the analyst.

### Procedure for unblinding if needed {17b}

The analyst will be unblinded after completion of analyses, to allow researchers to evaluate the results of the trial more thoroughly by providing transparent insights into how the intervention performed.

## Data collection and management

### Plans for assessment and collection of outcomes {18a}

As previously stated, baseline data collection for this trial happened as part of Wave 1 data collection of the DASH cohort study, between October 2024 and February 2025, while endline data collection will happen during Wave 2 data collection of the DASH cohort study, approximately 12 months after baseline. In order to measure MVPA and other physical activity outcomes described in the outcomes section, a physical activity questionnaire was developed by readapting questions from the Physical Activity Questionnaire for Adolescents (PAQ-A) [26], and the International Physical Activity Questionnaire for Adolescents (IPAQ-A) [27]. The questionnaire was then added to the overall DASH cohort study annual survey, which assesses a variety of health domains, including nutrition and physical activity, mental health, and sexual and reproductive health. Regarding physical activity, the questionnaire asks adolescents whether they engaged in any MVPA over the 7 days preceding the interview. If participants respond “yes,” they are asked how many days they participated in MVPA and the duration of MVPA on the last day. These questions cover both in-school and out-of-school physical activity.

The measure for intention to do physical activity is based on the Health Action Process Approach model [39] and is also embedded in the overall DASH questionnaire. It is a categorical variable asking if participants intend to change the amount of PA they will do in the coming week, compared to the 7 days preceding the interview. Participants can state whether they intend to do more PA, the same amount, or less. At baseline, all questions related to skipping rope outcomes were asked prior to providing the rope and pamphlet to participants during the interview, except the question on intention to do physical activity, which was asked after provision of the materials. The study questionnaire is available upon request.

### Plans to promote participant retention and complete follow-up {18b}

The fieldworkers provided participants with clear and detailed information on study goals, procedures, and benefits linked to participation, to increase their understanding of the benefits of physical activity and, in turn, their interest in skipping ropes. Participants do not receive incentives for participation or reminders during the 12-month study period.

### Data management {19}

All data are collected using electronic data collection forms on secure tablets and uploaded to a secure AAPH study server at UKZN, which is password-protected and encrypted, and can only be accessed by authorized personnel. Data sharing between institutions follows established secure protocols including encrypted file transfer and de-identification procedures. The data collection forms include validations to check the data. Only authorized study team members are granted access to electronic information about study participants by the study principal investigator (PI). Authorized team members regularly review data quality and integrity during the data collection process. As part of the implementation of the DASH cohort study, the study team at UKZN has access to identifiable data. For this trial, however, collaborators in the larger study team will only use the de-identified dataset processed and provided for the purpose of analysis and transmitted through secure means.

### Confidentiality {27}

All electronically collected data are automatically uploaded to a secure AAPH server at UKZN. Data is entered into electronic forms on secure tablets used by authorized study personnel. Participant identifiable information is stored separately from the rest of the participant data, and a key linking the two is stored in a separate third location at the site. The identifiable data is only accessible by the study team in South Africa. Data received by other teams will be de-identified and stored in an appropriate secure data storage space.

### Plans for collection, laboratory evaluation and storage of biological specimens for genetic or molecular analysis in this trial/future use {33}

NA: No biological specimens will be collected, evaluated, or stored for this trial.

## Statistical methods

### Statistical methods for primary and secondary outcomes {20a}

All statistical analyses will be conducted using Stata statistical software [40]. We will follow the CONSORT guidelines for reporting trial results [41].

#### Descriptive analysis

Socio-demographic characteristics of participants at baseline as well as at endline assessment will be compared between the two arms. The characteristics will be summarized using mean and standard deviation, median and interquartile range, or counts and proportions as appropriate. A simple eyeballing technique will be used to identify any imbalance in the two arms at baseline, and any potential imbalance will be addressed in the analysis. Descriptive analysis will also be used to describe process indicators.

#### Effectiveness analysis

The primary analysis will be intention-to-treat at the end of the trial. We aim to assess the effect of the treatment allocation (provision of skipping rope and informational pamphlet vs. control) on primary and secondary outcomes. For this, we will use linear regression models to analyze the difference in duration of MVPA per week between control and intervention groups at endline. In such a linear regression model, the coefficient of interest can be interpreted directly, i.e., in this analysis, it will yield the difference between the two groups in the number of minutes of MVPA per week. We will use logistic regressions for categorical secondary outcomes, including intention to do physical activity, and will report odds ratios. We will adjust for sex and other covariates that might not be balanced at baseline, as appropriate. A detailed statistical plan is included in the Supplementary Information (Additional file 2) as part of this protocol.

### Interim analyses {21b}

NA: No interim analyses will be conducted.

### Methods for additional analyses (e.g., subgroup analyses) {20b}

We will also conduct an analysis of outcomes stratified by sex, to assess whether the intervention has a different impact among boys versus girls. If appropriate, we might also stratify analyses by other physical, social, and emotional factors that might affect physical activity, such as BMI, depression, or anxiety. However, given that the study will not be well-powered to detect such an effect, this will be considered as exploratory.

### Methods in analysis to handle protocol non-adherence and any statistical methods to handle missing data {20c}

The research team will debrief with the fieldworkers to assess the extent to which they implemented the intervention as prompted by ODK and will closely monitor the completeness of data. In the case of missing values, the team will evaluate the number and type of missing data and will describe why data is missing. In case less than 10% of data will be missing, we will create a “missing” category for dichotomous variables. If more than 10% of data will be missing, we will perform multiple imputations (starting with approximately 20 imputations), and will use auxiliary variables that may include core socio-demographic variables, or variables linked to household and family, behavior and lifestyle, and anthropometry.

### Plans to give access to the full protocol, participant level-data and statistical code {31c}

There are ethical concerns about data privacy and confidentiality that require careful consideration before sharing individual-level data as the trial is embedded with the large DASH project. The final decision to make the data and codes available will lie with the DASH Investigators and participating institutions.

## Oversight and monitoring

### Composition of the coordinating center and trial steering committee {5d}

The main coordinating center for the trial is UKZN, in South Africa. Researchers at UKZN are working closely with fieldworkers and collaborators at the USINGA HDSS site to coordinate study activities, including recruitment and data collection. Collaborators at TUM, Heidelberg University, AAPH, and Harvard University will provide support regarding study design and data analysis. All teams will communicate regularly throughout the project to monitor study progress and ensure the integrity and confidentiality of data.

### Composition of the data monitoring committee, its role and reporting structure {21a}

NA: No data monitoring committee is formed for the trial. The teams at UKZN and AAPH will primarily be responsible for data monitoring.

### Adverse event reporting and harms {22}

In the unlikely scenario that an adverse event occurs, such as a physical injury related to skipping rope, it would be identified by the field team either throughout the study or during the endline survey. In order to monitor adverse events during the study, participants were instructed to report any issues to the research team, whose contact could be found on the consent forms. The event would then be reported to the site PI, who would in turn notify the PIs at other institutions, according to Good Clinical Practice guidelines. The investigators will submit a written notification of adverse events to the relevant Institutional Review Boards (IRBs) of Heidelberg University and UKZN. All adverse events will be documented with details of onset, duration, severity, outcome, and assessment of relatedness to the study intervention.

### Frequency and plans for auditing trial conduct {23}

NA: There is no plan for auditing trial conduct.

### Plans for communicating important protocol amendments to relevant parties (e.g., trial participants, ethical committees) {25}

In case of important protocol amendments, these will be communicated to the IRBs at Heidelberg University and UKZN in a timely manner.

### Dissemination plans {31a}

We will publish peer-reviewed articles on the study results and disseminate our findings at scientific meetings and conferences. Additionally, we will disseminate the results to stakeholders, including adolescents, their parents, policy makers, and funding agencies.

## Discussion

Physical activity is a critical component of a healthy lifestyle for adolescents, providing many benefits related to cardiovascular health, bone strength, flexibility, social and psychological well-being, and cognitive abilities [5]. Nevertheless, with the recent increase in sedentary lifestyles, adolescents in South Africa tend to be largely physically inactive [31]. This trial is a two-arm parallel-group, individual 1:1, unblinded randomized trial with the objective to test whether the provision of simple physical activity equipment and information on physical activity (intervention group) can improve physical activity-related intentions and behaviors, compared to no intervention (control group). Specifically, the primary objective of the trial is to test whether providing a skipping rope and informational pamphlet with information on the benefits of physical activity will increase the total amount of weekly MVPA done by adolescents, compared to not providing these materials.

The trial’s results will guide future research on physical activity and inform public health initiatives on physical activity in South Africa and other SSA countries. Additionally, the findings will hold significant implications for policymakers, particularly due to the low costs associated with providing skipping ropes and informational pamphlets. Skipping ropes are widely regarded as an accessible means of promoting physical activity among adolescents in South Africa. If the trial demonstrates positive outcomes in enhancing physical activity levels, policymakers should consider implementing this strategy across communities and schools. This could include ensuring that skipping ropes become a widely available tool in schools or community-based platforms, such as religious or youth centers, so that adolescents can have continued access to them throughout their youth, with potential for building life-long healthy physical activity habits.

This trial has several strengths. Traditionally, physical activity interventions in South Africa have focused on schools [42–45], often neglecting vulnerable out-of-school adolescents. In contrast, this trial will be implemented at the community level, effectively reaching both in-school and out-of-school adolescents. Furthermore, the trial will gather physical activity data from multiple settings in South Africa—in-school vs. out-of-school—offering valuable insights into differences in activity levels across these environments. The approach will be non-invasive, requiring only the provision of simple, low-cost equipment and information. However, the trial also has some limitations. Firstly, physical activity data will be self-reported rather than objectively measured, which may impact the reliability of the results. To enhance accuracy, participants will only be asked to recall their physical activity over the week leading up to the interviews. Additionally, given the nature and timeline of the DASH study, outcome measurements are only possible at baseline and 12 months after that, not allowing for outcome measurements in between waves, or after 12 months.

This skipping rope intervention will provide valuable insights into low-cost strategies to improve physical activity levels of adolescents in South Africa. If results show benefits associated with the skipping rope and pamphlet, policy makers should consider implementing such an intervention in communities and schools at large, as well as in other SSA countries.

## Trial status

Protocol version number 1, November 19th 2024.

Protocol version number 2, August 14th 2025.

Protocol version number 3, October 8th 2025.

Protocol version number 4, April 18th 2026.

Study recruitment begun on October 1 st 2024 and ended on February 28th 2025.

## Supplementary Information

Additional file 1. Informational Pamphlet on Skipping Rope and Physical Activity, Word document.

Additional file 2. Statistical Analysis Plan, Word document.

Additional file 3. Model Consent Form, PDF document.

Additional file 4. Ethical Approval Document from UKZN, PDF document.

Additional file 5. Funding Documentation, PDF document.

## Data Availability

Study forms will be available upon request. There are ethical concerns about data privacy and confidentiality that require careful consideration before sharing individual-level data as the trial is embedded with the large DASH project. The final decision to make the data and codes available will lie with the DASH Investigators and participating institutions.

## Abbreviations

DASH: Design and Evaluation of Adolescent Health Interventions and Policies in Sub-Saharan Africa
MVPA: Moderate-to-vigorous physical activity
WHO: World Health Organization
HDSS: Health and Demographic Surveillance System
USINGA: Umlazi Surveillance Initiative to Nurture Grassroots Action
PAQ-A: Physical Activity Questionnaire for Adolescents
IPAQ-A: International Physical Activity Questionnaire for Adolescents
ODK: Open Data Kit
IRB: Institutional Review Board
UKZN: University of Kwazulu-Natal
AAPH: Africa Academy for Public Health
