# Supplementary material for: Skipping rope and pamphlet intervention to promote physical activity among young adolescents in South Africa: study protocol for a randomized controlled trial
Source: Trials. 2026 May 11;27:362. doi: 10.1186/s13063-026-09752-x (PMC13162402; doi:10.1186/s13063-026-09752-x)
Supplement: Supplementary file 1 — Additional file 1. Informational Pamphlet on Skipping Rope and Physical Activity, Word document. [file 13063_2026_9752_MOESM1_ESM.docx]

**Physical Activity Pyramid**

This pyramid is a graphical representation of the different levels of physical activity that young people of your age should do in their daily lives.


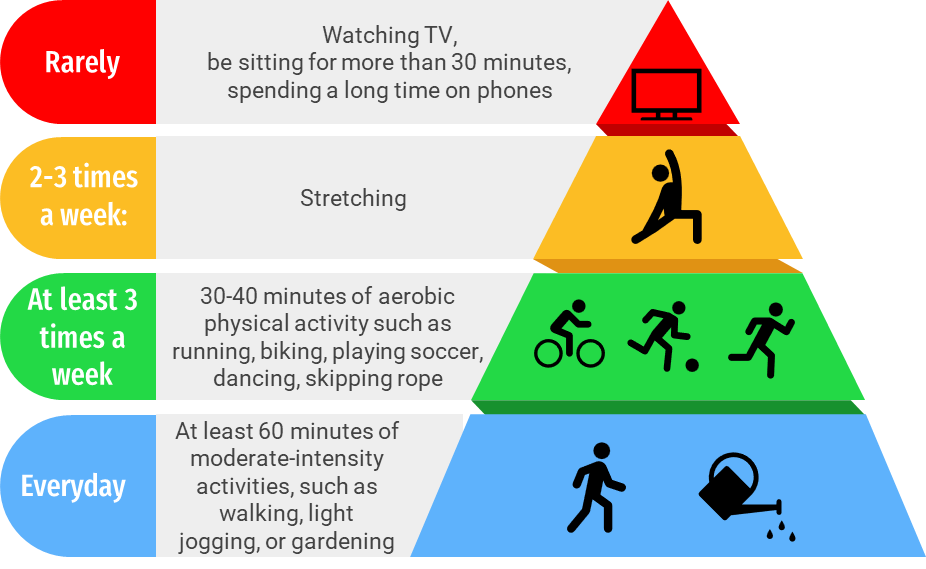


**Get Active With Skipping Rope!**

Start by skipping rope by yourself or with friends for **10-15 minutes every other day**. Mix it up with other fun activities. When it feels easier, aim to skip rope **for 10-15 minutes every day**.


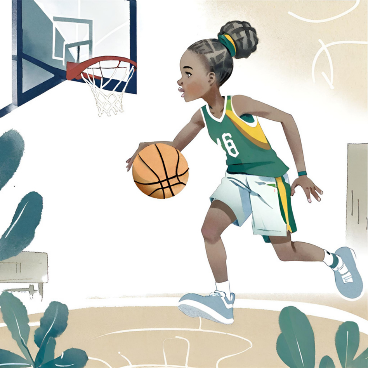
Aim for at least **60 minutes of physical activity every day**. Besides skipping rope, you can:

Go on fast walks, Jog, Dance, Bike, or Play sports with friends.

**Have fun and keep moving!**

5

**
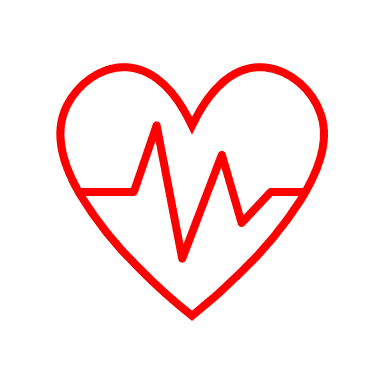
**Being physically active is only one aspect of a healthy lifestyle. Maintaining a healthy diet is also very important for your health. It is also important to get enough sleep so you can perform better at school the next day.


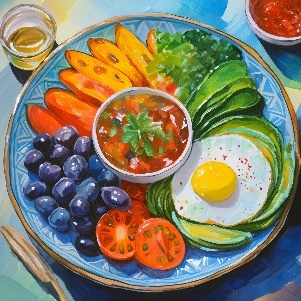

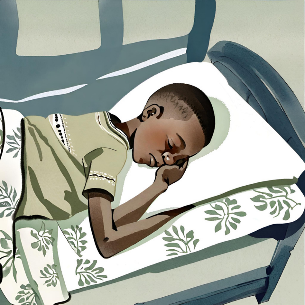


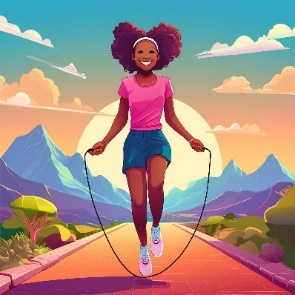


**If you have any questions, please contact [name of institution] at [phone number]**

**
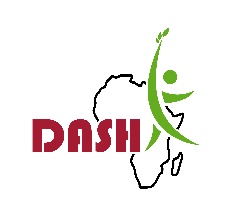
**

6

**Skipping Rope and Physical Activity**


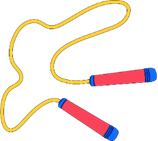
**Informational Pamphlet**

Physical activity is very important for people of your age because it helps you be healthy and grow well.

Skipping rope is an easy and fun way to be active! Boys and girls of your age often really enjoy this game.


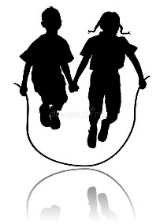

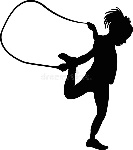

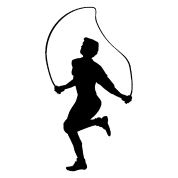

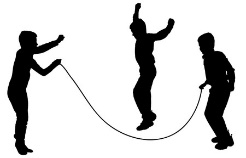
How to skip:

1. Ensure the rope reaches nearly up to your shoulders when folded in half.
2. Step over the rope, so that it hangs behind you.
3. Swing the rope over your head.
4. When the rope is coming towards the top of your feet, hop over it. Set a pace that works for you and repeat.
5. Play with your friends too!


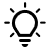
Start by practicing skipping rope by yourself or with your friends for **10-15 minutes every other day**. Once it becomes easier, **try to skip rope for 10-15 minutes every day.** Practicing is the key to getting good at it. **Get creative and have fun!**


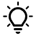
In total, **try to do physical activity for at least 60 minutes a day each day of the week.** In addition to skipping ropes, you can go on fast walks, jogs, dance, bike, or play a sport with your friends.

1

**Skipping Rope**

**
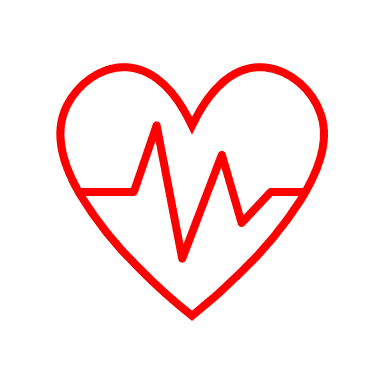
**Skipping rope has many benefits:

- Improves heart health
- Builds strong bones and muscles
- Enhances coordination and agility
- Boosts mood, self-confidence, and energy levels
- Increases fitness to do other sports better
- Is fun to do with friends and family

Skipping rope is used by people at all ages to stay fit.


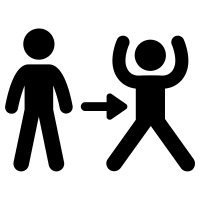
**
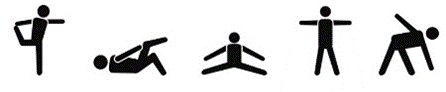
**
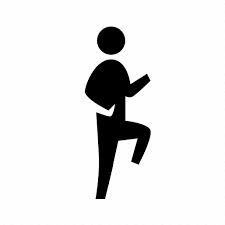
**Remember to warm up.** Warming up prior to skipping is an important step to prevent you from getting injured and prepare your body for skipping the rope. Ideas for warmups include but are not limited to:

**
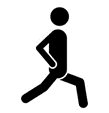

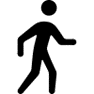
**

Simple stretches

High knees

Jumping jacks

Fast Walking

Lunges


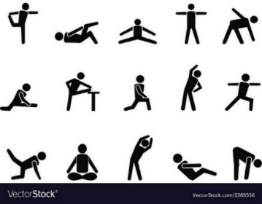
 **Lastly, stretch again at the end of the workout!** This will help your body cool down.


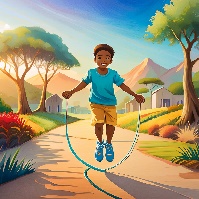

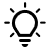


**Tricks to Change the Routine**

There are many ways to jump rope. Here are a few ideas:

- **Scissor jumps:** land with one foot forward and switch feet at the next jump.
- **Jump rope high knees:** switch feet at each jump and bring your knees as high as you can.
- **Reverse the rope:** jump the rope going backwards. Start with the rope in front of your feet and throw it over your head to the back. Jump over it before it hits the back of your feet.
- **Skier jumps:** using both feet, first jump to the left, then to the right.
- **Bell jumps:** jump forward, then backward using both feet. One jump per revolution of the rope.
- **Double dutch**: two people turn two ropes in opposite directions while a third person in the middle jumps over each rope. More thank one person can jump in the middle.
- **Combine the tricks and turn on music if you’d like!**

**Fun Fact!**

Did you know that skipping rope has been popular for centuries? Even the ancient Egyptians did it! Join people around the world and have fun skipping rope!


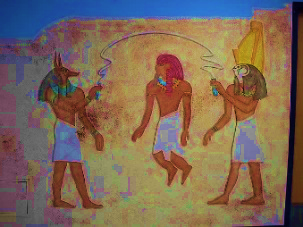


2

3

**Why Stay Physically Active?**


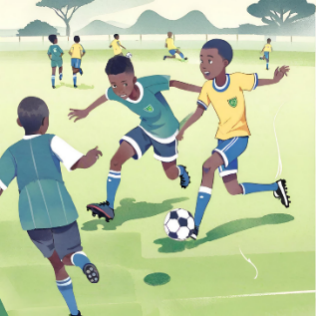
Being active is very important during adolescence. It helps you keep a healthy heart, body, and mind, and supports your growth. Physical activity means moving your body, and there are lots of fun ways to do it, like: walking, running, bicycling or playing team sports. **Find what you enjoy and get moving!**.

**How Much Physical Activity Should I Do?**

You should:

- Do at least an average of 60 minutes per day of moderate-to-vigorous intensity activity across the week.


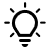


If you can **comfortably talk but not sing**, you are doing activity at **a** **moderate level**.

- Do vigorous-intensity activities at least 3 days a week.


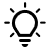
If you cannot say more than a few words **without breathing heavily**, you are doing activity at **a vigorous level.**

- Limit the amount of time spent sitting down and not moving.

4
