# Supplementary material for: Skipping rope and pamphlet intervention to promote physical activity among young adolescents in South Africa: study protocol for a randomized controlled trial
Source: Trials. 2026 May 11;27:362. doi: 10.1186/s13063-026-09752-x (PMC13162402; doi:10.1186/s13063-026-09752-x)
Supplement: Supplementary file 2 — Additional file 2. Statistical Analysis Plan, Word document. [file 13063_2026_9752_MOESM2_ESM.docx]

**Skipping Rope and Pamphlet Intervention to Promote Physical Activity among Young Adolescents in South Africa: Study Protocol for a Randomized Controlled Trial**

**Additional File 2**

**Statistical Analysis Plan**

**TABLE OF CONTENTS**

[LIST OF ABBREVIATIONS 3](#_Toc209797323)

[1. Study Objectives 4](#_Toc209797324)

[2. STUDY DESIGN 4](#_Toc209797325)

[3. STUDY POPULATION 4](#_Toc209797326)

[4. DESCRIPTION OF STUDY ACTIVITIES 4](#_Toc209797327)

[5. hypotheses 5](#_Toc209797328)

[6. Outcome Variables 5](#_Toc209797329)

[7. SAMPLE SIZE 5](#_Toc209797330)

[8. Populations of Analysis 5](#_Toc209797331)

[8.1. INCLUSION CRITERIA 5](#_Toc209797332)

[8.2. EXCLUISON CRITERIA 6](#_Toc209797333)

[8.3. ALLOCATION OF INTERVENTION 6](#_Toc209797334)

[9. HANDLING OF MISSING DATA 6](#_Toc209797335)

[10. Statistical Methodology 6](#_Toc209797336)

[10.1. DESCRIPTIVE ANALYSIS 6](#_Toc209797337)

[10.2. INFERENTIAL ANALYSIS 6](#_Toc209797338)

[10.3. MASKING 6](#_Toc209797339)

[11. References 7](#_Toc209797340)

[12. Tables 8](#_Toc209797341)

LIST OF ABBREVIATIONS

SSA Sub-Saharan Africa

VMPA Moderate-Vigorous Physical Activity

DASH Design and Evaluation of Adolescent Health Interventions and Policies

SD Standard Deviation

HDSS Health and Demographic Surveillance System

PAQ-A Physical Activity Questionnaire for Adolescents

IPAQ-A International Physical Activity Questionnaire for Adolescents

1. Study Objectives

The study aims to investigate the effect of providing a skipping rope and an informational pamphlet with physical activity information (intervention group) on physical activity intentions and behaviors among adolescents in South Africa, compared to no intervention (control group). We hypothesize that providing the skipping rope and informational pamphlet about physical activity to adolescents will be more effective in improving their physical activity behaviors at 12 months after baseline assessment, compared to not providing these materials.

1. STUDY DESIGN

This study comprises a parallel-group, individual 1:1, randomized controlled trial nested within an adolescent cohort, i.e. a ‘randomized trial-within-cohort’. The intervention includes providing each participant with a skipping rope and a pamphlet with general instructions for skipping the rope and messages about the importance of physical activity. The materials were distributed during the baseline data collection (Wave 1) of the DASH cohort study, with outcome assessment occurring at baseline for intention to do physical activity and at endline for changes in physical activity behaviours.

1. STUDY POPULATION

DASH is a research network for the design and evaluation of adolescent health interventions and policies in SSA (1). The overall goal of this project is to boost adolescent health in SSA through rigorous population-based intervention and policy research. The skipping rope intervention is being implemented as part of DASH in the Umlazi Health and Demographic Surveillance System (HDSS) in south Africa, also called USINGA (Umlazi Surveillance Initiative to Nurture Grassroots Action). This HDSS operates as a standardized and comprehensive field-based information system and research platform. It collects prospective data from entire populations, including adolescents, at both individual and household levels, from communities facing developmental constraints (2).

As part of DASH, a random sample of 2000 individuals aged 10-24 was drawn from existing population sampling frames created and maintained by the USINGA HDSS, to ensure that the population is fully representative of the community. This DASH cohort will be followed up over four waves of data collection. Of these 2000 individuals, all those aged 10-14 were invited to participate in this randomized trial.

1. DESCRIPTION OF STUDY ACTIVITIES

This randomized trial will assess the effect of providing the skipping rope and pamphlet with physical activity information (intervention group) compared to no intervention (control group) on measures of physical activity intentions and behaviours among adolescents. Each of these intervention conditions is described in more detail below.

**Intervention group**

The provision of rope and informational pamphlet to the intervention group happened during the participant interview within the overall DASH cohort study. The fieldworker conducting the survey interview provided each participant in the intervention group with a rope and pamphlet and read a short script explaining how to use the rope, outlining the main benefits of physical activity for adolescents, and asking participants to read the pamphlet in their own time.

**Control group**

The control group did not receive any intervention.

1. hypotheses

The primary hypothesis is that providing a skipping rope and informational pamphlet about physical activity to adolescents will be more effective in improving their levels of moderate-vigorous physical activity (MVPA) at 12 months after baseline assessment, compared to not providing these materials.

The secondary hypotheses are that providing rope and pamphlet to adolescents will also increase the proportion of adolescents who meet the MVPA recommendations over the 7 days preceding the interview at 12 months after baseline assessment, compared to not providing these materials. Additionally, providing these materials will increase the number of adolescents who intend to engage in physical activity over the 7 days following the interview, at baseline assessment, compared to not providing these materials.

1. Outcome Variables

Data will be collected at two timepoints - baseline and 12-months - and will be based on self-reported physical activity. Questions have been readapted from the Physical Activity Questionnaire for Adolescents (PAQ-A), and the International Physical Activity Questionnaire for Adolescents (IPAQ-A). The variables are described in Table 1.

1. SAMPLE SIZE

For the overall DASH cohort study, a random sample of 2000 individuals aged 10-24 were drawn from existing population sampling frames created and maintained by the Umlazi HDSS in South Africa. Of these 2000 individuals, all those aged 10-14 were invited to participate in this randomized trial.

**Power calculation**

We have approximately 700 participants enrolled. To calculate the statistical power for our intervention trial, we used a Stata menu-driven command to estimate power based on various scenarios for the changes in primary outcome, total minutes of MVPA/week. The total minutes of MVPA per week are based on previous research on physical activity for adolescents in South Africa and a few other countries (3–11). Based on such research, the following assumptions are considered:

- Minutes of MVPA per week among adolescents in South Africa vary significantly. To be conservative, we assume a baseline value of 250 min/week of MVPA across boys and girls, with a standard deviation (SD) of 160 min/week (3).
- 15% loss to follow-up.

Based on these assumptions, with 700 participants and 80% power, the proposed trial will detect a minimum difference of 33.9 min/week in MVPA between the intervention and comparison arms, with 160 min/week SD and 350 participants in each arm (Table 2).

1. Populations of Analysis
   1. INCLUSION CRITERIA

Inclusion criteria for this study are as follows:

- Study participant is between ages 10 and 14 years;
- Minors (i.e. less than 18 years of age): one of the parents or a guardian provides written informed consent;
- Minors: the minor participant provides informed assent; and,
- Study participant is a resident of the study area and intends to stay in the study area for the duration of the study.
  1. EXCLUISON CRITERIA

The following exclusion criteria will be applied to the study:

- Participants who do not meet the above-listed criteria;
- Participants with physical disabilities that prevent them from doing physical activity ;
- Those participants whose capacity to make meaningful decisions is in question because they are "cognitively impaired”;
- Individuals with communication difficulties; and,
- Study participants who report suicidal behaviours during baseline data collection.
  1. ALLOCATION OF INTERVENTION

As described above, this trial is nested within the DASH cohort study, which uses existing population sampling frames in its sampling strategy. Using the pre-defined sampling roster for that study, researchers used a statistical computing software to define a random allocation sequence for all potentially eligible participants (i.e. those aged 10-14 years). This was a simple 1:1 random allocation. The fieldworkers were not involved in the sequence generation.

1. HANDLING OF MISSING DATA

In the case of missing values, the team will evaluate the number and type of missing data and will describe why data is missing. In case less than 10% of data will be missing, we will create a “missing” category for dichotomous variables. If more than 10% of data will be missing, we will perform multiple imputations (starting with approximately 20 imputations), and will use auxiliary variables that may include core socio-demographic variables, or variables linked to household and family, behaviour and lifestyle, and anthropometry.

1. Statistical Methodology
   1. DESCRIPTIVE ANALYSIS

Socio-demographic characteristics of participants at baseline as well as at endline assessment will be compared between the two arms. The characteristics will be summarized using mean and standard deviation, median and inter-quartile range, or counts and proportions as appropriate. Eyeballing technique will be used to identify any imbalance in the two arms at baseline, and any potential imbalance will be addressed in the analysis. Descriptive analysis will also be used to describe process indicators.

- 1. INFERENTIAL ANALYSIS

The primary analysis will be intention-to-treat at the end of the trial. We aim to analyze the effect of the treatment allocation (provision of skipping rope and informational pamphlet vs. control) on primary and secondary outcomes. For this, we will use linear regression models to analyze the difference in duration of MVPA per week between control and intervention groups at endline. In such a linear regression model, the coefficient of interest can be interpreted directly, i.e. in this analysis, it will yield the difference between the two groups in the number of minutes MVPA per week. We will use logistic regressions for categorical secondary outcomes, reporting odds ratios. We will adjust for sex and other covariates that might not be balanced at baseline, as appropriate.

- 1. MASKING

The analyst, or outcome assessor, will be masked in this clinical trial and will not know allocation status until analysis is complete.

1. References

1. Heidelberg University. DASH: Research Network for Design and Evaluation of Adolescent Health Interventions and Policies in Sub-Saharan Africa [Internet]. [cited 2024 Aug 30]. Available from: https://www.klinikum.uni-heidelberg.de/heidelberger-institut-fuer-global-health/groups-projects/research-projects/dash

2. South African Population Research Infrastructure Network (SAPRIN) [Internet]. [cited 2024 Jun 4]. Available from: https://saprin.mrc.ac.za/usinga.html

3. Wushe SN, Moss SJ, Monyeki MA. Objectively determined habitual physical activity in South African adolescents: The PAHL study. BMC Public Health. 2014 May 19;14(1).

4. Corder K, Brown HE, Schiff A, Van Sluijs EMF. Feasibility study and pilot cluster-randomised controlled trial of the GoActive intervention aiming to promote physical activity among adolescents: outcomes and lessons learnt. BMJ Open [Internet]. 2016;6. Available from: http://dx.doi.org/10.1136/bmjopen-2016-012335

5. Neto AS, Dos Santos GC, Da Silva JM, Correa RC, Da Mata LBF, De O. Barbosa R, et al. Improving physical activity behaviors, physical fitness, cardiometabolic and mental health in adolescents - ActTeens Program: A protocol for a randomized controlled trial. PLoS One. 2022 Aug 1;17(8 August).

6. Healthy Active Kids South Africa - Report Card. 2022.

7. Dolley D, Du Randt R, Pühse U, Gerber M, Bosma J, Aerts A, et al. Relationship between Body Mass Index and Physical Activity among Children from Low-Income Communities in Gqeberha, South Africa: A Cross-Sectional Study. Int J Environ Res Public Health. 2023 Jan 1;20(2).

8. Mcveigh J, Meiring R. Physical Activity and Sedentary Behavior in an Ethnically Diverse Group of South African School Children [Internet]. Vol. 13, ©Journal of Sports Science and Medicine. 2014. Available from: http://www.jssm.org

9. Hanson SK, Munthali RJ, Micklesfield LK, Lobelo F, Cunningham SA, Hartman TJ, et al. Longitudinal patterns of physical activity, sedentary behavior and sleep in urban South African adolescents, Birth-To-Twenty Plus cohort. BMC Pediatr. 2019 Jul 18;19(1):241.

10. Micklesfield LK, Pedro TM, Kahn K, Kinsman J, Pettifor JM, Tollman S, et al. Physical activity and sedentary behavior among adolescents in rural South Africa: levels, patterns and correlates [Internet]. 2014. Available from: http://www.biomedcentral.com/1471-2458/14/40

11. Jemmott JB, Zhang J, Jemmott LS, Icard LD, Ngwane Z, Makiwane M, et al. Intervention Increases Physical Activity and Healthful Diet Among South African Adolescents Over 54 Months: A Randomized Controlled Trial. Journal of Adolescent Health. 2019 Jul 1;65(1):139–46.

1. Tables

**Table 1. Outcome Variables***

| **Primary Outcomes** |  |  |
| --- | --- | --- |
| **Outcome** | **Description** | **Specifications and Timeline** |
| Weekly Moderate-Vigorous Physical Activity | Continuous outcome of total minutes of MVPA/week | Calculated by multiplying the minutes of MVPA on the most recent day participant did MVPA, by the number of days MVPA was done. Will be assessed separately for in- and out-of-school MVPA, and the total duration will be obtained by summing the two.  Assessed at endline (12 months after baseline) |
| **Secondary Outcomes** |  |  |
| **Outcome** | **Description** | **Specifications and Timeline** |
| Intention to engage in physical activity over the 7 days following the interview, compared to the 7 days preceding it | Categorical outcome of intention to engage in physical activity | Assessed at baseline |
| Meeting the WHO MVPA recommendations over the 7 days preceding the interview | Categorical binary outcome of whether adolescents meet the WHO MVPA recommendations of at least 60 minutes/day of MVPA across the week (>=420 minutes/week) | Assessed at endline (12 months after baseline) |
| **Process Outcomes** |  |  |
| **Outcome** | **Description** | **Measures and Specifications** |
| Intervention group: modality of skipping | Categorical outcome for modality of skipping rope | Assessed at endline (12 months after baseline) |
| Control group only: Rope sharing | Categorical outcome for frequency of rope sharing | Assessed at endline (12 months after baseline) |

*For all outcomes, the source is the physical activity section of the DASH Questionnaire.

**Table 2. Power calculation for the randomized controlled trial for MVPA outcome (n= 700 participants, 350 participants in each arm)**

| The table shows that with a power of 80%, 350 participants in each arm will yield a minimum detectable difference of 33.9, with a baseline mean value of 250 minutes/week of MVPA and SD of 160 minutes/week. The number of participants per arm may vary. | | | | |
| --- | --- | --- | --- | --- |
| Baseline Mean | Standard Deviation | Minimum detectable difference in mean | Number of participants in each arm | Expected power |
| 250 | 160 | 43.7 | 350 | 95% |
| 250 | 160 | 39.3 | 350 | 90% |
| 250 | 160 | 36.3 | 350 | 85% |
| 250 | 160 | 33.9 | 350 | 80% |
| 250 | 160 | 31.9 | 350 | 75% |
