# Supplementary material for: Skipping rope and pamphlet intervention to promote physical activity among young adolescents in South Africa: study protocol for a randomized controlled trial
Source: Trials. 2026 May 11;27:362. doi: 10.1186/s13063-026-09752-x (PMC13162402; doi:10.1186/s13063-026-09752-x)
Supplement: Supplementary file 3 — Additional file 3. Model Consent Form, PDF document. [file 13063_2026_9752_MOESM3_ESM.pdf]

UKZN BIOMEDICAL RESEARCH ETHICS COMMITTEE

APPLICATION FOR ETHICS APPROVAL
For research with human participants (Biomedical)

STUDY INFORMATION SHEET

Title of the study: DASH – Research network for design and evaluation of adolescent health interventions and policies in Sub-Saharan Africa

---

Principal Investigator: Prof. Mosa Moshabela

University of KwaZulu-Natal, Deputy Vice Chancellor, Research and Innovation

Email address: Moshabela@ukzn.ac.za

This study is funded by GIZ

Greetings,

We thank you for your interest in hearing about our study. In this information sheet you will find important information about the study we would like you to participate in, as well as further information about study procedures, activities, data processing and security, risks, and benefits for you. Please read the following information carefully. You can then decide whether you would like to participate or not. Feel free to take your time and to ask the study staff any questions about issues you do not understand or which are important to you.

What are the objectives of this research?

This study aims to deliver a better understanding of behaviours that may affect nutrition, mental health and sexual and reproductive health in adolescents, and to explore how these areas of health could be improved. It will also test whether interventions focused on these behaviours can improve the physical and mental well-being of adolescents. To achieve this, we are inviting young people, like yourself, to take part in the study and you have been selected by chance to participate.

Do I have to take part in this study?

Your participation in this study is **voluntary,** meaning you do not have to take part in the study if you do not wish to and you can decide to withdraw from the study at any point.

During all activities, such as during answering a questionnaire, you can pause or end at any time, and if you prefer not to answer a question you can always choose to skip it. There are no “right” or “wrong” answers and you can decide to withdraw from the study. Refusal to participate or withdrawal from the study will not involve negative consequences for you. The study will take place over four years and each year we will contact you and will ask you to confirm that you wish to continue in the study before completing the annual survey.

How does the study work?

The study contains two activities and your participation in this study may include either being invited to take part in each activity described below or even just the annual survey. Preceding the study, we ask you to read this participant information sheet and sign the informed consent form.

The study is being conducted in seven African countries (Tanzania, Ghana, Burkina Faso, Nigeria, Uganda, Ethiopia and South Africa). In the sections below we describe each of the activities you may be invited to participate in.

ACTIVITY 1: Survey

All participants will be invited to take part in the first part of the study to participate in an interview to complete a survey. These surveys will involve questions about your health, diet, physical activity, and other topics important for your health, such as mental health, sexual practices, smoking and consuming alcohol. At the end of the interviews your height and weight will be measured, and we will conduct a finger prick test to take a drop of blood, and use a device which will measure your haemoglobin concentration and allow us to determine whether you may have anaemia. The survey takes, on average, between 60 to 90 minutes.

You will be invited to take part in the survey each year for a period of four years. The height and weight measurements and the finger prick test will also be done each year.

The data collected in the survey will be kept confidential and will not be shared with anyone beyond the study team.

You can find more information on privacy and data security below.

ACTIVITY 2: Interventions

As part of the study, we will provide interventions that will focus on providing you educational materials on nutrition, mental health or sexual and reproductive health – depending on the intervention. It is possible that you may be selected to take part in an intervention, but you also may not be selected to take part in one. Who receives them, and who does not, will be decided by chance and the study team has no control over this and around half of the participants will be selected. If you are not selected to receive an intervention, by the end of the study you will have the opportunity to receive all study materials (such as, for example, pamphlets or videos) including information on areas such as nutrition, healthy eating, hygiene practices and prevention of anaemia, as well as on mental health and sexual and reproductive health.

If you are selected to take part we will contact you and provide further information on the intervention you have been selected for and you can decide to participate or not.

At the end of the study, we will hold a local dissemination event for all participants in the study to learn more about the results and conclusions of the study.

What are the risks?

There is a risk that some participants may feel uncomfortable, stressed or anxious talking about some of the topics included in the survey. However, we do not wish for this to happen. You do not have to answer any questions that you do not feel comfortable responding to, and you may at any time choose to skip a question or end the interview without giving any reason. The annual survey and the individual and group discussions will take on average around one-hour which may be a burden for some participants.

Blood collection from the finger bears the risk of pain, and potential bleeding from the puncture site. Also, temporary dizziness or fainting may occur. In rare cases, infection may result. In very rare cases, taking blood from the vein may result in nerve damage. During the anthropometric measurements participants may have to remove excess clothes (such as a sweater) and take off shoes which some participants may find inconvenient or unpleasant. During the course of the study you may receive information that could be distressing such as that you are anaemic or are overweight.

What are the benefits?

We cannot promise you will receive any direct benefits from taking part in this study. However, the measurements we will make during the physical examination, which may be

important to you, will be explained clearly to you. If needed, we will refer you to seek additional care and/or counselling.

If you are selected, by chance, to receive an intervention, you will be provided with educational and potentially nutritional supplements in addition to the materials. It is possible that receiving this intervention could influence your behaviour and health in a positive way, but we do not know that for sure.

Additionally, the information derived from this study will help to inform those people and institutions that make decisions on policies that affect the health of adolescents and young adults. Therefore, the results of this study have the potential to improve adolescent health in your community and in others.

If I take part in this study, how will my privacy be protected? What happens to the information you collect?

By signing the informed consent form, you declare that you agree that the principal investigator and team members collect and process your personal data for the purpose of the study described above. Personal data are, for example, name, birth date and address. This data will be stored electronically and will be secured against unauthorized access.

The principal investigators will use your personal data for purposes of administration and study conduct such as in the event of the withdrawal of the study for the purpose of destroying the data. Before your data are analysed, they will be given a pseudonym – meaning that the collected data are marked with a number/letter code instead of the name/date of birth, etc so that your name is no longer in the data. The code key, which connects this pseudonym to your personal identity, will only be accessible to the principal investigators and study team members. The code key will be destroyed as soon as possible after completion of data evaluation and the data will be completely anonymous – meaning that your data cannot be identified by anyone.

Information sharing between partners in the study will adhere to the data sharing and transfer agreement regulations of the European Union and respective institutions/countries. The anonymous data may be shared publicly, however this means that no information with personal identifying data will be shared with other parties outside of the study.

You have the right to request and receive all personal data concerning yourself in a structured, commonly used and legible format and have the right to transfer this data to another data controller. You also have the right to correct any incorrect personal data. You

can also request that your data be deleted and we will delete it as soon as possible or to stop processing or using some, or all, of your personal data.

In these cases, and if you have any questions concerning the study or data being collected, please contact the study team or the principal investigators. You can find their address and phone number below. You can withdraw your agreement to have your data collected and processed for this study **at any time**. Please also note that the results of this study may be published in the scientific literature. However, this will be done in a way so that it is impossible to trace any results to you.

Organizations that may inspect and have access to your pseudonymized information include the other representatives of the organizations/institutions involved in this study, including:

- Universitätsklinikum Heidelberg (UKHD)
- Technische Universität München (TUM)
- Centre De Recherche En Santé De Nouna (CRSN)
- University of Dodoma (UDOM)
- Addis Continental Entrepreneurs PLC (ACE)
- Africa Academy for Public Health (AAPH)
- University of Ibadan Research Foundation (UI)
- Makerere University (MUK)
- University of Ghana (UG)
- University of KwaZulu-Natal (UKZN)
- Harvard Global Research and Support Services Inc. (Harvard)

Right of withdrawal:

You are free to withdraw from this study **at any time and for any reason**, **without any negative consequences**. If you choose to withdraw, you can choose whether you want the information collected so far to be used or not. You also have the option to request that any previously collected information be destroyed. You are welcome to change your mind about these choices at any time in the future.

If you have any questions, please don't hesitate to ask the interviewer, who will be happy to answer them for you. You can also request clarification or ask additional questions at any point during the study.

Compensation and costs:

By participating in this study there will be no costs for you. You will not receive any payment to take part in the study.

In the event of any problems or concerns/questions you may contact the Principal Investigator at 031-260 2381 or the UKZN Biomedical Research Ethics Committee, contact details as follows:

BIOMEDICAL RESEARCH ETHICS ADMINISTRATION
Research Office, Westville Campus
Govan Mbeki Building
Private Bag X 54001
Durban
4000
KwaZulu-Natal, SOUTH AFRICA
Tel: 27 31 2602486 - Fax: 27 31 2604609
Email: BREC@ukzn.ac.za

UKZN BIOMEDICAL RESEARCH ETHICS COMMITTEE

APPLICATION FOR ETHICS APPROVAL
For research with human participants (Biomedical)

INFORMED CONSENT FORM

DASH - Research Network For Design And Evaluation Of Adolescent Health Interventions And Policies In Sub-Saharan Africa Study Informed Consent

I hereby declare that I have been informed about all details of the above-mentioned study and that I have been given the opportunity to clarify my questions with the study team.

I have been informed and voluntarily consent that the data collected from me in this study, in particular information about my health, will be stored, evaluated and, if necessary, potentially forwarded to the respective member institutions/countries listed in the information sheet in pseudonymous form for the purposes described in the information leaflet. Third parties will not be given access to personal data at any time. My name will not be disclosed when the results of the study are published.

The personal data will be anonymized as soon as this is possible according to the research purpose. The data will be destroyed after completion of data evaluation.

I am aware that this consent can be revoked at any time in writing or verbally without giving reasons, without that I will suffer any disadvantages as a result. The legality of the data processing carried out up to the revocation of data processing is not affected by this. In this case, I can decide whether the data collected from me should be deleted or may continue to be used for the purposes of the study.

I have been informed and voluntarily consent to the evaluation and processing of information about my health, ethnic origin and sexual behavior, for the purposes described in the informational document in pseudonymized form. I have received a copy of the written participant information sheet and the consent form.

I declare that I voluntarily agree to participate in this scientific study. I agree that:

1. **My personal data that are necessary for the purpose of the above-mentioned study can be collected by the study team member on behalf of the principal investigators and are stored and processed in a pseudonymised manner.**
2. **The study team is allowed to take notes during the study interview to ensure information is not lost.**
3. **The study results may be published in anonymous form which does not allow any conclusions on my person.**
4. **For the purpose of the above-mentioned study my pseudonymised data can be shared with respective member institutions/countries in the study after following the necessary data transfer and sharing processes.**

....

....

Place, Date Signature of participant

Statement of person witnessing consent (Process for Non-Literate Participants):

I.. (Name of Witness) certify that information given to

.... (Name of Participant), in the local language, is a true reflection of what l have read from the study Participant Information Leaflet, attached.

WITNESS' SIGNATURE (maintain if participant is illiterate): ……………………………………….

Hereby, I declare that I have informed in oral and written form the participant on the date of ……………............................ about nature, meaning, and risks of the above-mentioned study, that I have answered all questions, and that I have handed over copies of the Participant Information Form and the Informed Consent Form over to the study participant.

....

....

Place, Date Name and signature of informing study team member

UKZN BIOMEDICAL RESEARCH ETHICS COMMITTEE

APPLICATION FOR ETHICS APPROVAL
For research with human participants (Biomedical)

ASSENT FORM

DASH - Research network for design and evaluation of adolescent health interventions and policies in Sub-Saharan Africa

I hereby, confirm that a study team member explained to me in detail what this study is about and in which activities I will be participating in if I decide to be in the study. I had the possibility to ask questions about the study and my participation in it and, if I had questions, received answers that helped me understand it better. I confirm that I was explained that it is my choice whether or not to be in the study, and that I can change my mind anytime if I decide that I don't want to be in the study anymore without anyone being angry or upset with me.

With my signature below, I agree that I am willing to participate in this study.

....	....
Place, Date	Signature of participant

Statement of person witnessing consent (Process for Non-Literate Participants):

I………………………………………….   (Name of Witness) certify that information given to

.... (Name of Participant), in the local language, is a true reflection of what l have read from the study Participant Information Leaflet, attached.

WITNESS’ SIGNATURE (maintain if participant is non-literate): …………………………………….

Hereby, I declare that I have informed in oral and written form the participant on the date of ……………….......................... about nature, meaning, and risks of the above-mentioned study, that I have answered all questions, and that I have handed over copies of the Participant Information Form and the Informed Consent Form over to the study participant.

....	....
Place, Date	Name and signature of informing study team member
