# Supplementary material for: Skipping rope and pamphlet intervention to promote physical activity among young adolescents in South Africa: study protocol for a randomized controlled trial
Source: Trials. 2026 May 11;27:362. doi: 10.1186/s13063-026-09752-x (PMC13162402; doi:10.1186/s13063-026-09752-x)
Supplement: Supplementary file 5 — Additional file 5. Funding Documentation, PDF document. [file 13063_2026_9752_MOESM5_ESM.pdf]

Skipping Rope and Pamphlet Intervention to Promote Physical Activity among Young Adolescents in South Africa: Study Protocol for a Randomized Controlled Trial

Funding Documentation

The current document provides information regarding funding for DASH – Network for Design and Evaluation of Interventions and Policy on Adolescent Health in Sub-Saharan Africa. The Skipping Rope trial is nested within the DASH project. Funding information can be found from:

1. The German Federal Ministry of Education and Research (BMBF) website, which is the study funder. This website provides information on study aims, total amount of funding, and funding period for the two German partners, who are involved in the project: https://www.gesundheitsforschung-bmbf.de/de/dash-netzwerk-fur-design-und-evaluation-von-interventionen-und-politik-zur-16369.php

   Website translation (can also be done directly on web through Google Translate):

   DASH – Network for Design and Evaluation of Interventions and Policy on Adolescent Health in Sub-Saharan Africa

   The aim of DASH is to promote youth health in Sub-Saharan Africa. In the African partner countries, intervention measures and the corresponding existing political framework in the areas of nutrition and physical activity, sexual and reproductive health, mental health and violence are to be evaluated and improved. The planned research activities include the identification of health risk factors and the local need for intervention, the design, development and piloting of promising interventions as well as their evaluation and transfer into policy and practice. This is intended to close important research gaps in the need for intervention measures and their design, impact and transferability in the area of youth health.

   Subprojects

   Method development and evaluation of interventions and health policies

Funding code:	01KA2219A
Total funding amount:	1,053,794 EUR
Funding period:	2023 - 2028
Project management:	Prof. Dr. Michael Laxy
Address:	Technical University of Munich, Faculty of Sport and Health Sciences<br>Georg-Brauchle-Ring 60/62<br>80992 Munich

The overarching and long-term goal of the 'Network for the Design and Evaluation of Interventions and Policies for Adolescent Health in Sub-Saharan Africa' (DASH) is to promote adolescent health in Sub-Saharan Africa through population-based intervention and policy research. To achieve this goal, a

combination of efficient collection and utilisation of relevant adolescent health data and the use of robust quantitative and qualitative methods with local, domain-specific expertise in the areas of nutrition and physical activity, sexual and reproductive health, mental health and violence will be used. Through the planned research activities, DASH has the potential to significantly strengthen the research infrastructure and evidence base and thus also the health of young people in Sub-Saharan Africa. The aim is to close important research gaps in the need for intervention and policy measures and their design, impact, performance and transferability. In addition to the research agenda, investments will be made in activities and relationships that ensure sustainability and societal impact. Here the focus is on capacity building within and outside the network and networking and collaboration with young people and their parents, other researchers and policy makers. The Technical University of Munich (TUM) will lead the scientific investigation of the effects, impact and mechanisms of key national and regional health policies and co-lead the development of a methodological approach for researching interventions to promote adolescent health in Sub-Saharan Africa. In addition, TUM will contribute to the networking work package and support the planning and implementation of all work packages.

Improving sexual and reproductive health

Funding code: 01KA2219B

Total funding amount: 1,355,165 EUR

Funding period: 2023 - 2028

Project management: Prof. Dr. Till Bärnighausen

Address: Heidelberg University Hospital, Medical Faculty, Institute of Public Health
Im Neuenheimer Feld 324
69120 Heidelberg

The overarching and long-term goal of the 'Network for the Design and Evaluation of Interventions and Policies for Adolescent Health in Sub-Saharan Africa' (DASH) is to promote adolescent health in Sub-Saharan Africa through population-based intervention and policy research. To achieve this goal, a combination of efficient collection and utilisation of relevant adolescent health data and the use of robust quantitative and qualitative methods with local, domain-specific expertise in the areas of nutrition and physical activity, sexual and reproductive health, mental health and violence will be used. Through the planned research activities, DASH has the potential to significantly strengthen the research infrastructure and evidence base and thereby also the health of adolescents in Sub-Saharan Africa. The aim is to close important research gaps in the need for intervention and policy measures and their design, impact,

performance and transferability. In addition to the research agenda, investments will be made in activities and relationships that ensure sustainability and societal impact. Here the focus is on capacity building within and outside the network and networking and collaboration with young people and their parents, other researchers and policy makers. The Heidelberg Institute of Global Health of Heidelberg University (HIGH) will lead the research on adolescent health interventions to improve sexual and reproductive health and co-lead the development of a methodological approach for research on interventions to promote adolescent health in Sub-Saharan Africa. The HIGH will also contribute to the capacity building work package and support the planning and implementation of all work packages.

2. The DASH Network website, which explains more about the Network and provides information about the sponsor: https://www.dash-rhissa.org/
