# Supplementary material for: Skipping rope and pamphlet intervention to promote physical activity among young adolescents in South Africa: study protocol for a randomized controlled trial
Source: Trials. 2026 May 11;27:362. doi: 10.1186/s13063-026-09752-x (PMC13162402; doi:10.1186/s13063-026-09752-x)
Supplement: Supplementary file 4 — Additional file 4. Ethical Approval Document from UKZN, PDF document. [file 13063_2026_9752_MOESM4_ESM.pdf]

UNIVERSITY OF KWAZULU-NATAL™
INYUVESI YAKWAZULU-NATALI

25 May 2024

Prof Moshabela, Matlagolo Mosa (27896)
Dr Jabulani Ncayiyana (504474)
School of Nurs & Public Health

Dear Prof Moshabela and Dr Ncayiyana,

Protocol reference number: BREC/00006459/2023
Project title: DASH - Research Network for Design and Evaluation of Adolescent Health Interventions and Policies in Sub-Saharan Africa
Non-degree

FULL COMMITTEE APPROVAL LETTER

The Biomedical Research Ethics Committee (BREC) has considered the abovementioned application at a meeting held on 12 December 2023.

I wish to advise you that your response to BREC correspondence dated 12 January 2024 was noted and approved by a sub-committee of the Biomedical Research Ethics Committee. Please ensure that any outstanding site permissions are obtained and forwarded to BREC for approval before commencing research at a new site.

This approval is valid for one year from **25 May 2024.** To ensure uninterrupted approval of this study beyond the approval expiry date, an application for recertification must be submitted to BREC on RIG before the expiry date.

Any amendments to this study, unless urgently required to ensure safety of participants, must be approved by BREC prior to implementation.

Your acceptance of this approval denotes your compliance with South African National Research Ethics Guidelines (2015), South African National Good Clinical Practice Guidelines (2020) (if applicable) and with UKZN BREC ethics requirements as contained in the UKZN BREC Terms of Reference and Standard Operating Procedures, all available at http://research.ukzn.ac.za/Research-Ethics/Biomedical-Research-Ethics.aspx.

BREC is registered with the South African National Health Research Ethics Council (REC-290408-009). BREC has US Office for Human Research Protections (OHRP) Federal-wide Assurance (FWA 678).

Biomedical Research Ethics Committee
Chair: Professor S Singh
UKZN Research Ethics Office Westville Campus, Govan Mbeki Building
Postal Address: Private Bag X54001, Durban 4000
Email: BREC@ukzn.ac.za
Website: http://research.ukzn.ac.za/Research-Ethics/Biomedical-Research-Ethics.aspx

Founding Campuses: Edgewood Howard College Medical School Pietermaritzburg Westville

INSPIRING GREATNESS

The following Committee members were present at the meeting that took place on 12 December 2023:

Prof D Wassenaar	Clinical Psychology and Research Ethics (Chair)
Prof R Bhimma	Paediatrics & Child Health (Deputy Chair)
Prof C Aldous	Genetics and Research Support
Ptr S D Chili	Community/Lay Member (External)
Prof T Hardcastle	Surgery
Dr A Harrichandparsad	Public Health
Dr R Harrichandparsad	Neurosurgery
Dr H Humphries	Research Psychology and Public Health
Dr M Khan	HIV Clinician
Dr Z Khumalo	KZN Health, General Medicine (External)
Dr R Lessells	Infectious Diseases Specialist
Prof D Moodley	Women's Health
Dr S Paruk	Psychiatry
Dr P Pillay	Clinical Anatomy
Dr H Ramnarain	Obstetrics and Gynaecology
Dr D Singh	Critical Care
Dr M Smith	Anaesthetics and Critical Care
Prof A Strode	Health Law and Research Ethics

We wish you well with this study. We would appreciate receiving copies of all publications arising out of this study.

This approval will be noted at the next BREC meeting to be held on 11 June 2024.

Yours sincerely,

PROFESSOR S SINGH
Chair: Biomedical Research Ethics Committee
